# Assessing Future Maladaptation Risk in the Keystone Species *Populus tremuloides*

**DOI:** 10.1111/eva.70332

**Published:** 2026-10-13

**Authors:** Melanie Zacharias, Roos Goessen, Christian Wehenkel, Benjamin Marquis, Chen Ding, Fred Raley, Jerritt Nunneley, Nathalie Isabel, Ilga Porth

**Affiliations:** ^1^ Institute for System and Integrated Biology (IBIS), Université Laval Québec Québec Canada; ^2^ Département de biologie chimie et géographie, Université de Québec à Rimouski Rimouski, Québec Canada; ^3^ Instituto de Silvicultura e Industria de la Madera, Universidad Juarez del Estado de Durango Durango Mexico; ^4^ Laurentian Forest Centre, Canadian Forest Service, Natural Resources Canada Québec Québec Canada; ^5^ Département de Science et Technologie Université TÉLUQ Montréal, Québec Canada; ^6^ College of Forestry, Wildlife and Environment, Auburn University Auburn Alabama USA; ^7^ Western Gulf Forest Tree Improvement Program, Texas A&M Forest Service College Station Texas USA; ^8^ Texas Biomedical Research Institute San Antonio Texas USA

## Abstract

Understanding the adaptive potential of widespread trees is critical for predicting species resilience under climate change and informing their conservation strategies. We assessed the genomic offset in *Populus tremuloides* Michx. (aspen) using 10,937 genotyping‐by‐sequencing derived, high‐quality SNPs from 891 individuals sampled across the species' range, from Mexico to both the eastern and western regions of North America. Gradient forest models were applied using multiple SNP datasets and climate scenarios, with the genomic offset standardized by aspen population genetic clusters and ecological regions to identify future habitat suitability (recipient importance) and potential sources of reproductive material (donor importance) for assisted migration. Moisture‐related variables exerted consistently stronger selective pressure on genetic variation than temperature, reflecting the riparian ecology of aspen. Populations from Mexico and the southwestern U.S.A. exhibited the highest offsets, suggesting elevated vulnerability despite apparent drought adaptations, whereas northern populations showed the lowest offsets, consistent with projected northward range shifts. Traits not captured by genomic offset models, such as polyploidy, clonality, hybridization, and epigenetics, may buffer environmental stress and enhance resilience, underscoring the limitations of purely genomic predictions. To complement offset estimates, we tested linear models using phenotypic data (growth, polyploidy), but the limited representation of aspen populations in common garden trials restricted validation. Nevertheless, a positive association between polyploidy and offset emerged, suggesting a potential adaptive or buffering mechanism that warrants further investigation. Together, our results illustrate both the promise and the limitations of genomic offset as a predictive framework for widely distributed species. By presenting the first approach to scale the genomic offset to a broadly‐distributed forest tree, this study provides a foundation for integrating genomic, phenotypic, and ecological data to inform climate‐resilient forest management. Range‐wide common garden and provenance trials remain essential, yet they remain scarce for broadleaf species such as aspen. Integrating multiple data sources is critical for developing effective climate‐resilient forest management strategies.

## 1 Introduction

Currently, our planet is experiencing profound climatic change that will continue, as global surface temperatures are projected to rise by 1.4°C to 4.4°C by 2100 (Intergovernmental Panel on Climate Change 2023). Traditionally, populations of organisms living in natural ecosystems are believed to be the best adapted to their local environments, as natural selection favors individuals with traits that are well‐suited to local conditions (Linhart and Grant 1996). However, growing evidence suggests that long‐lived species such as trees may be unable to adapt quickly enough to keep pace with climate change (Aitken et al. 2008; Dauphin et al. 2021), potentially challenging the *status quo* of the “local is best” paradigm.

Climate change has introduced a temporal mismatch, whereby tree populations are better adapted to the historical climate conditions in which they established, sometimes decades or centuries ago, than to current or projected future conditions (Hamann et al. 2013; Rehfeldt et al. 2012). Emerging novel climates across landscapes only further complicate predictions of species responses (Mahony et al. 2017). This intergenerational adaptation lag calls into question conventional seed sourcing strategies and supports considering climate‐adjusted provenancing approaches such as assisted migration (Bower et al. 2024).

Assisted migration has gained attention as a strategy to enhance the adaptive capacity and resilience of forest ecosystems (Aitken and Bemmels 2016; Z. Chen, Grossfurthner, et al. 2022; Simler et al. 2019; Twardek et al. 2023; Williams and Dumroese 2013). It involves intentionally moving individuals either within the species' current range (assisted gene flow) or beyond it (assisted species migration). Assisted gene flow focuses on transferring genotypes carrying alleles pre‐adapted to anticipated future conditions, thereby increasing genetic diversity and buffering against climatic instability (Aitken and Whitlock 2013). While assisted migration offers benefits, such as enhancing adaptation potential and reducing extinction risk, it also carries risks like invasiveness, outbreeding depression, or ecological disruption, which have been thoroughly discussed (Aitken and Whitlock 2013; Aubin et al. 2011; McLachlan et al. 2007; Williams and Dumroese 2013; Xu and Prescott 2024) along with implementation guidelines (Bower et al. 2024; Stanturf et al. 2024).

Some countries already integrate climate adaptation into reforestation practices. For example, Australia combines local and non‐local material seed sourcing strategies to enhance genetic diversity and climate resilience (Hancock et al. 2023). In Germany, sourcing includes multiple populations within a region to reduce maladaptation occurrence (Bucharova et al. 2019). Furthermore, Canada established an assisted migration trial in southern Ontario to test the northward movement of six broadleaf and the southward movement of three conifer species. Results indicated that both broadleaf and coniferous provenances tolerated significant relocation while still achieving acceptable growth and survival rates (Pedlar et al. 2024). Several Canadian provinces have already adjusted their seed transfer guidelines. British Columbia and Alberta have expanded transfer zones to higher elevation areas for most species, with Alberta also extending them northward, while Québec has begun planting mixed seed sources to reduce the risks of climate maladaptation (Government of Canada 2025).

To support such approaches, the highest‐risk populations for future maladaptation must be identified, as well as populations that possess the highest adaptive potential under future climate conditions. Evaluating adaptive potential in a location‐specific context is essential, particularly when assessing suitability for future planting sites. For economically important species, such as white spruce, common garden trials (provenance‐progeny trials, clonal trials, etc.) provide a robust basis for evaluating adaptive potential and site suitability at specific locations where the trials were established. For species lacking these extensive phenotypic datasets, which are the majority of species, one promising alternative is calculating the genomic offset using machine learning approaches like Gradient Forest (Ellis et al. 2012), which has gained traction in recent years (Gougherty et al. 2021; Gugger et al. 2021; Lind et al. 2024). Originally developed to model species community turnover across landscapes (Ellis et al. 2012), Gradient Forest (GF) has since been adapted for landscape genomics using SNP data to model allele frequency shifts along environmental gradients (Fitzpatrick and Keller 2015). This approach enables calculating the genomic offset, defined as the genetic mismatch between current allele frequencies and those predicted under future climate scenarios (Rellstab 2021). Also referred to as genomic vulnerability or the risk of non‐adaptedness, the genomic offset was determined for several tree species, including oaks (Gugger et al. 2021; Martins et al. 2018; Vanhove et al. 2021), poplars (Gougherty et al. 2021), and cypress (Jia et al. 2020). Recent simulation studies confirmed that the genomic offset is a valuable predictor of fitness under environmental change (Lind and Lotterhos 2024; Rellstab and Keller 2025). It often outperforms predictions based solely on climate distances or ecological niche modeling (Y. Chen, Jiang, et al. 2022; Fitzpatrick et al. 2021; Sang et al. 2022) and has been associated with observed population declines (Bay et al. 2018; Ruegg et al. 2018), making it a potential tool for conservation and management planning. Nonetheless, genomic offset estimations face challenges, including the influence of population structure, migration, and gene flow (Gain et al. 2023). Gradient forest models assume that sampled populations are locally adapted and estimate the genomic offset as the mismatch between observed allele frequencies and those predicted under future climate scenarios. However, these predictions are inherently based on the current spatial genetic structure, which may arise from a combination of adaptive processes, demographic history, and neutral population structure, not all of which reflect local adaptation, particularly in species with high gene flow and clonality such as *P. tremuloides* . Consequently, in our study, we interpret genomic offset values as indicators of potential maladaptation risk under future climates given the current genomic landscape, rather than as measures of the adaptive capacity of populations. Moreover, the metric lacks standard units, complicating comparisons across studies, even within the same species (Lachmuth et al. 2023). Importantly, it assumes that local genotypes are the most fit, which may not universally hold (Jones 2013).

To address these limitations, Lachmuth et al. (2023) proposed a standardization procedure that rescales the genomic offset relative to current genetic differentiation. In addition, they introduced donor and recipient importance scores to assess which populations are best suited to provide or receive genetic material under future climates (Lachmuth et al. 2024). To validate this framework, linear models were tested to see whether higher genomic offset correlated with reduced growth performance, a proxy for maladaptation. The results of this and other studies revealed that the GF offset indeed correlates with lower fitness (Láruson et al. 2022; Lachmuth et al. 2024).

Given the species‐specific local adaptation and phylogeographic structure patterns (collectively, the genetic structure observed in a species), analyses of maladaptation risk must be conducted on a species‐by‐species basis. In this study, we focus on *Populus tremuloides* Michx. (quaking aspen), a widespread keystone species in North America that provides vital ecosystem services like food and shelter for several species or ecosystem regeneration (Perala et al. 1990). Aspen's distribution extends from the east to the west coast and into central Mexico, encompassing a broad range of climatic and environmental conditions. *P. tremuloides* is a dioecious species with high genetic diversity, even exceeding that of *P. trichocarpa* or *P. tremula* (J. Wang, Street, et al. 2016) and reproduces both sexually through seeds and asexually through root suckering (Barnes 1966). Its ability to rapidly colonize disturbed sites makes it a key early‐successional species that plays an important role in post‐disturbance regeneration (Swift and Ran 2012). As climate change is expected to increase fire frequency and severity across much of its range (Wang et al. 2025), aspen's fire resilience and rapid regrowth make it an attractive species for reforestation in disturbance‐prone landscapes (Shinneman et al. 2013). Given its broad geographic distribution and its ability to adjust and thrive across a wide range of environmental conditions, *P. tremuloides* serves as an ideal model species for evaluating how genomic‐offset‐based approaches can inform assisted migration and climate‐resilient reforestation strategies.

In this study, two key research questions are addressed:

- How can the genomic offset be effectively calculated and standardized for a widely distributed species to inform assisted migration strategies in the context of climate change?
- Which regions within the *P. tremuloides* distribution range are at the highest risk of future maladaptation, and which regions may serve as optimal source populations for assisted migration?

To answer these questions, we used a comprehensive genomic dataset containing 891 individuals representing the species' full range, including dry regions in Texas, mountainous regions in Mexico, and both coastal and continental populations. Using the R package GradientForest, we calculated the genomic offset under different climate scenarios. We adapted the standardized offset approach developed by Lachmuth et al. (2023) to account for current genetic differentiation and assessed donor and recipient importance across populations. To validate our predictions, we analyzed phenotypic data from common garden trials via linear models, testing whether populations with higher offset values showed reduced growth performance. Our approach demonstrates how the standardized genomic offset can be used to identify vulnerable populations and inform climate‐adaptive forest management for widely distributed species.

## 2 Material and Methods

### 2.1 Genetic Data

The genomic dataset used in this study was initially published by Goessen et al. (2022), comprising ~1000 individuals and 44,000 SNPs obtained by genotyping‐by‐sequencing methodology (GbS), and was later extended by Goessen et al. (2026) to include samples from across the full species range, including 150 additional individuals from Texas, U.S.A. DNA was extracted from dried leaf tissue as described in Goessen et al. (2022). Details on sampling design, GbS library preparation, and sequencing protocols can be found in Goessen et al. (2022); Goessen, Isabel, Wehenkel, et al. (2025).

SNP calling and filtering were performed using the STACKS‐based pipeline developed by Eric Normandeau at the Institute for System and Integrated Biology (IBIS), Université Laval, Québec, Québec, Canada (https://github.com/enormandeau/stacks_workflow), following the parameters 8 90 0 59 (minimum depth of 8, a percentage of SNP missingness of 90%), and a minimum MAS (minimum number of samples with rare allele) of 2.5% of the original number of samples (i.e., MAS of 59) and a relatedness threshold of 0.3 as detailed in Goessen et al. (2022) using the new *P. tremuloides* reference genome described in Goessen, Isabel, Wehenkel, et al. (2025). We excluded individuals with more than 10% missing data and removed SNPs with more than 20% missing data. After retaining only diploid individuals, the dataset comprised 891 samples and 13,033 SNPs, with a missing data rate of 1.76%.

To tailor the dataset to fit our analyses, we applied additional quality filtering. To reduce false‐positive associations, we filtered for a minor allele frequency (MAF) ≥ 0.05, following established recommendations (Gugger et al. 2018; Ruegg et al. 2018; Yu et al. 2022). Missing genotypes were imputed using the *na.roughfix* function from the R package randomForest v.4.7–1.1 (Breiman et al. 2002), which replaces missing values with the mean genotype per locus, a necessary step as Gradient Forest does not accommodate missing data. Only diploid, non‐clonal individuals were retained for analysis. The final dataset included 10,937 SNPs from 891 *P. tremuloides* individuals sampled across the species' range, from Mexico to both the eastern and western parts of North America (Figure 1).

**FIGURE 1:**
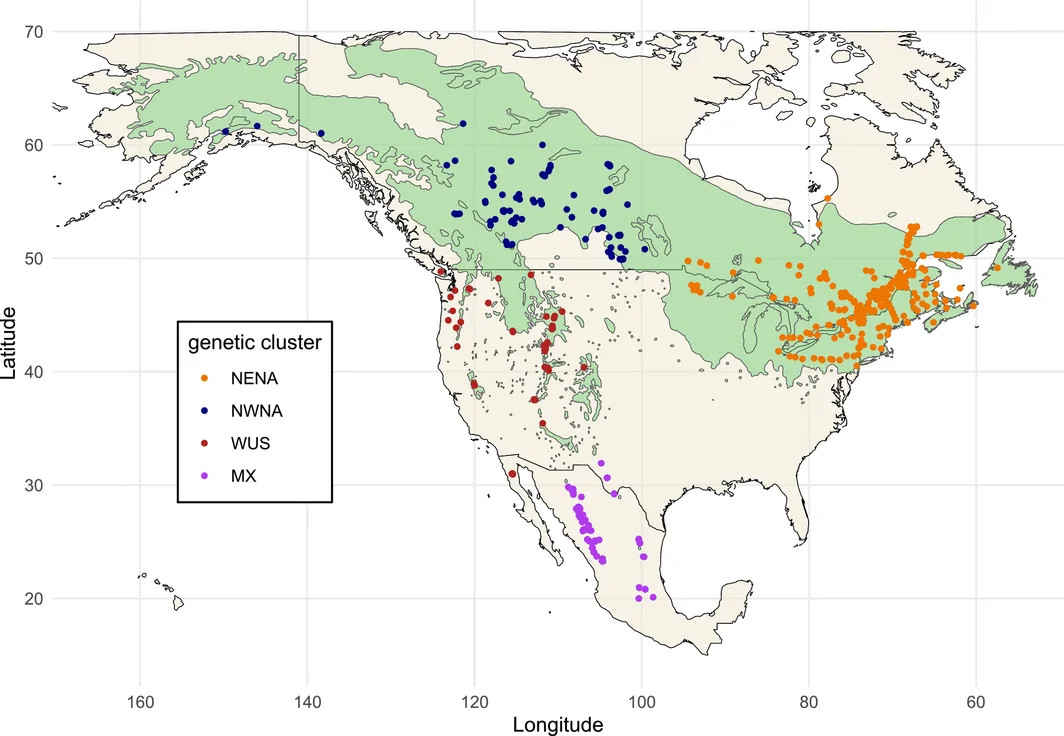
*Populus tremuloides* species distribution (green) across North America and sampled populations colored according to their genetic cluster (lineage) membership (Goessen, Isabel, Wehenkel, et al. 2025). Details are described in Table S1.

Goessen et al. (2022) identified four major genetic lineages across the *P. tremuloides* range: North‐Eastern North America (NENA), North‐Western North America (NWNA), the Western United States (WUS), and Mexico (MX) (Figure 1, Figure S1). While the NWNA and NENA lineages were genetically distinct, their pairwise *F*_ST_ values indicated relatively low genetic differentiation compared to the remaining aspen lineages. High triploidy and clonality were observed particularly within WUS and MX populations. Across all geographic regions, aspen populations displayed high genetic diversity, with most variation found within individuals, i.e., heterozygosity (Goessen et al. 2022). Complementary samples obtained from Goessen et al. (2026) and (Goessen, Isabel, Wehenkel, et al. 2025) were assigned to the existing four‐cluster structure established by the initial 2022 study (Figure 1, Table S1).

In line with recommendations by Lind and Lotterhos (2024), we assessed the sensitivity of Gradient Forest outputs by screening multiple genetic marker sets. Specifically, we used four types of datasets: (1) a full dataset containing the 10,937 SNPs, (2) a neutral dataset comprising 391 SNPs located in intergenic regions, (3) a candidate dataset of 283 SNPs identified via Bayenv2, and (4) a candidate dataset with 420 SNPs identified via LFMM. Bayenv2 and LFMM were selected because they identified SNPs associated with environmental variables, representing loci potentially under selection. Including these candidate SNPs allowed us to evaluate whether incorporating environmentally associated loci improved the predictive power of genomic offset models compared to those based on neutral or full SNP datasets.

The Bayenv2 SNPs were the same as those previously identified with the program Bayenv2 (Günther and Coop 2013) in Goessen, Isabel, Wehenkel, et al. (2025). LFMM SNPs were identified using the Latent factor mixed models 2.0 (Caye et al. 2019) within the R package LEA (Frichot and François 2015) as described in Goessen, Isabel, Wehenkel, et al. (2025): because LFMM2 does not allow missing data, we imputed missing genotypes using a script (developed by Manuel Lamothe, Natural Resources Canada, Laurentian Forestry Centre), which applies a machine learning approach implemented in the R package XGBoost (Chen and Guestrin 2016) with the following parameters: booster = “dart”, eta = 0.2, gamma = 0, subsample = 0.8, objective = “multi:softmax”, and nrounds = 200. LFMM2 was run with five latent factors, representing the four major lineages and additional substructure within the western U.S. lineage, based on the variance explained by each principal component as implemented in the LFMM2 package.

We utilized adjusted *p*‐values using the Benjamini‐Hochberg correction to reduce false‐positives. The resulting SNPs that showed an adjusted *p*‐value below 0.05 were deemed significantly associated with the environmental variables tested. Identified SNPs are listed in the supplements (“candidate_SNPs_Bayenv.csv”, “candidate_SNPs_LFMM.csv”). To avoid redundancy, SNPs overlapping between the candidate and neutral datasets were removed from the latter (12 Bayenv SNPs, 15 LFMM SNPs). The two candidate datasets overlapped in 111 SNPs, which were kept in both datasets.

### 2.2 Climate Data

We used historical climate data obtained from ClimateNA v7.00 (T. Wang, Hamann, et al. 2016), representing the 30‐year average for the period 1961–1990 (Table 1). To capture broad‐scale climatic patterns across the large latitudinal gradient of *P. tremuloides* , we applied a spatial resolution of 20 × 20 km grid cells. This resolution was chosen to emphasize macroclimatic variation from south to north rather than microclimatic differences. Additionally, using smaller grid cells across the species' extensive distribution would have significantly increased computational demand.

**TABLE 1:** ClimateNA variables used in the model.

Abbreviation	Climate variable
MAT	Mean annual temperature (°C)
MWMT	Mean warmest month temperature (°C)
MCMT	Mean coldest month temperature (°C)
TD	Temperature difference (MWMT—MCMT), or continentality (°C)
MAP	Mean annual precipitation (mm)
MSP	Mean summer precipitation (mm) (May–September)
AHM	Annual Heat‐Moisture Index (MAT +10)/(MAP/1000)
SHM	Summer Heat‐Moisture Index (MWMT)/(MSP/1000)
DD_0	Degree‐days below 0°C
DD5	Degree‐days above 5°C
DD_18	Degree‐days below 18°C
DD18	Degree‐days above 18°C
NFFD	Number of frost‐free days
FFP	Frost‐Free Period
bFFP	Beginning of frost‐free period (Day of Year)
eFFP	End of frost‐free period (Day of Year)
PAS	Precipitation as snow (mm) (August–July)
EMT	Extreme minimum temperature over 30 years (°C)
EXT	Extreme maximum temperature over 30 years (°C)
Eref	Hargreaves reference evaporation (mm)
CMD	Climatic moisture deficit (mm)
RH	Mean annual relative humidity (%)
CMI	Hogg's Climate Moisture Index (mm)
DD1040	Degree‐days between 10°C and 40°C

To project the genomic offset under future climate conditions, we used three Shared Socioeconomic Pathway (SSP) scenarios that represent different levels of greenhouse gas emissions and associated warming. Specifically, we selected climate projections for the time period 2041–2070 under the following SSPs: SSP2‐4.5, representing moderate warming of approximately 2.5°C–3.0°C; SSP3‐7.0, representing more severe warming of 3.5°C–4.0°C, and SSP5‐8.5, representing the most extreme scenario with projected warming of 4.0°C–5.0°C (Hamann et al. 2013; Mahony et al. 2021). These future climate projections were used as inputs to calculate the genomic offset and assess the potential maladaptation risk for *P. tremuloides* populations across the entire distribution range.

### 2.3 Gradient Forest Model and Genomic Offset Calculation

In this study, we determined the genomic offset within the species *P. tremuloides* , based on the foundational assumption that populations are locally adapted to their environments. This assumption is critical for validating the genomic offset analyses, as emphasized by Lind and Lotterhos (2024), who highlighted the need to demonstrate local adaptation prior to applying such analytical models. Evidence for local adaptation in *P. tremuloides* was first provided by Goessen et al. (2022); Goessen, Isabel, Wehenkel, et al. (2025) and also by Hernández‐Velasco et al. (2025), who detected loci putatively under selection by climatic variables, indicating geographically divergent adaptive evolution among populations. Their findings support the idea that *P. tremuloides* populations are adapted to distinct environmental conditions, thereby justifying the use of genomic offset in this system (Hernández‐Velasco et al. 2025).

To model genotype‐environment relationships, we used the R package gradientForest v.0.1–37 (Ellis et al. 2012), fitting 5000 regression trees (ntree = 5000) per SNP, while keeping all other parameters at default settings. The dependent variables were the different SNP datasets described earlier (full, neutral, Bayenv candidates, and LFMM candidates), and the independent variables were the climate predictors. After fitting the model, the trained Gradient Forest (GF) was used to predict genomic composition across the entire *P. tremuloides* distribution range. These GF predictions reflect the multidimensional genomic variation structured along climate gradients.

Because neutral population structure can confound genotype‐environment associations and cannot be explicitly accounted for within the GF framework, we incorporated spatial structure into the climate data matrix using Moran's Eigenvector Maps (MEMs) (Fitzpatrick and Keller 2015; Rellstab 2021). We calculated MEM axes 1–5 using the *mem* function from the adespatial package (Dray et al. 2012) and added them to the environmental predictor matrix to account for spatial autocorrelation.

To visualize the modeled genomic variation, we performed a Principal Component Analysis (PCA) using the *prcomp* function from the R stats package. Following Fitzpatrick and Keller's (2015) and Yu et al. (2022) approaches, we used the first three principal components to define an RGB color palette, which was applied in biplots and to map GF‐predicted genomic variation across the species' range. Similar colors indicate similar genomic composition among regions. Based on variable importance scores from the GF model, we selected a six‐key climate variables subset which represented the most influential climate variables. For highly correlated climate variables (*r* > 0.8), only one was retained in the analysis to reduce multicollinearity. For further analysis, we selected: temperature difference between the warmest and coldest month (TD), May to September precipitation (MSP), the summer heat‐moisture index (SHM), reference evapotranspiration (Eref), climatic moisture deficit (CMD), and the climate moisture index (CMI).

The genomic offset was calculated using the GF model as the Euclidean distance between the predicted present‐day genomic composition and the composition required under future climate conditions. This represents the magnitude of allele frequency shifts necessary to remain adapted in a changing environment. Future climate projections used in this calculation are described in the section “Climate Data.”

### 2.4 Standardizing Genomic Offset and Assessing Donor/Recipient Importance for *P. tremuloides* ' Wide Distribution Range

To standardize the genomic offset, we employed the method developed by Lachmuth et al. (2023) using the corresponding R package offsetEnsembleR v.0.90. Among the tested models, the GF model built using LFMM candidate SNPs explained the highest proportion of genomic variation (Table 2), although differences among SNP sets were modest. We therefore selected this model for downstream analyses not solely based on statistical performance, but because LFMM identifies genotype‐environment associations while accounting for latent population structure, thereby enriching for loci more likely involved in climate‐related selection. Given that our objective is to inform management under future climates, a dataset enriched for putatively adaptive variation provides a more biologically meaningful basis for estimating maladaptation risk. Nonetheless, we note that quantitative estimates of donor or recipient importance may differ under alternative SNP sets, including neutral loci, even if qualitative spatial patterns are broadly consistent. To maintain analytical consistency and focus on an intermediate climate trajectory, we conducted offset projections under the SSP3‐7.0 scenario only.

**TABLE 2:** SNPs selected to build the GF models.

Dataset types	SNPs	Selected SNPs model	Mean *R* ^2^ (%)	Overlap with full dataset
Candidate Bayenv2	283	138 (49%)	17	136
Candidate LFMM	420	316 (75%)	22	314
Full	10,937	3192 (29%)	16	—
Neutral	391	101 (26%)	18	95

When standardizing the genomic offset, Lachmuth et al. (2023) employed GF models using allele frequencies instead of genotypes. A recent study, however, showed that using genotypes over allele frequencies improves model accuracy (Lind and Lotterhos 2026). We therefore used the LFMM GF model as described previously. All offset calculations were performed using functions from the offsetEnsembleR package. We began by calculating contemporary spatial offsets among all currently occupied grid cells using the *spatOffset* function, based on the transformed environmental predictors (turnover functions) from the GF model. This function computes an empirical cumulative distribution function (ECDF), accounting for current genetic differentiation and potential maladaptation among populations. Next, we calculated spatio‐temporal offsets using the *spatTempOffset* function, which measures the mismatch between present‐day genomic composition and that predicted under future climate scenarios, while allowing for potential spatial movement of populations like assisted migration. As future environmental input, we used the SSP3‐7.0 scenario (see section “Climate Data”). To increase interpretability, we scaled the spatio‐temporal offsets using the *standardizeOffset* function, following the standardization approach described in Lachmuth et al. (2023, 2024). This method expresses spatio‐temporal offsets as probabilities relative to the spatial offsets calculated previously, allowing for a biologically meaningful genomic mismatch interpretation. We further applied quantile normalization to convert the scaled offsets into z‐scores, enabling standardized comparisons across populations and aiding the ecological interpretation of shifts in gene–environment association space. Finally, we calculated donor and recipient importance using the *donor.recipientImportance* function. This step evaluates the genomic offset between current donor populations and potential recipient locations under future conditions across the full *P. tremuloides* distribution. Following Lachmuth et al. (2023), we applied a maladaptation threshold of 1 standard deviation (1*σ*) and excluded donor–recipient pairs exceeding this threshold. Among acceptable matches, additional calculations included: recipient importance, defined as the proportion of donor cells (= donor populations) able to immigrate without exceeding the maladaptation threshold, indicating how broadly a recipient site may accept genetic material under future climate conditions; donor importance, defined as the proportions of recipient locations to which where a given donor population could be transferred without exceeding the threshold, reflecting the donor's potential utility across the landscape. For full methodological details, see Lachmuth et al. (2023, 2024).

Given *P. tremuloides* ' vast distribution, long‐distance transfers, such as from Mexico to Alaska, are ecologically unrealistic. To provide a more practical framework for assessing genetic transfer potential, we divided the North American distribution into latitudinal ecological zones based on the FAO ecological region classifications. We defined three ecozones: Boreal (50°–70° N), Temperate (40°–50° N), and Subtropical (20°–40° N). Within each zone, offsets and donor/recipient importance were recalculated independently to provide region‐specific recommendations for assisted migration. In addition to the species' wide geographic range, *P. tremuloides* exhibits pronounced population structure (Goessen et al. 2022; Goessen, Isabel, Wehenkel, et al. 2025). As suggested by Susanne Lachmuth (personal communication), offset standardization should be performed within genetic clusters to account for the underlying genetic differentiation. To incorporate this, we ran GF models for each of the four known genetic lineages in aspen as previously identified by Goessen et al. (2022), and we standardized and analyzed offsets separately. To assign each 20 × 20 km grid cell to a genetic cluster, we implemented a k‐nearest neighbors (KNN) classification using the *knn* function from the R package class v.7.3–23 (Ripley 2009). Grid cells populated with populations of known cluster assignments were used as the training set, and grid cells without a cluster assignment were used as the test set. The cluster assignment of unclassified grid cells was based on distances calculated from the transformed climate predictors in the GF model, using the aforementioned allele frequencies of the LFMM candidate SNPs. For each genetic cluster, we recalculated spatial and spatio‐temporal offsets using only grid cells within that cluster, ensuring that ECDFs used for standardization were localized and reflective of cluster‐specific variation. The same pipeline was applied, calculating spatio‐temporal offsets with *spatTempOffset*, standardizing with *standardizeOffset*, and computing donor and recipient importance within clusters using *donor.recipientImportance*, to produce more accurate, population‐specific recommendations for assisted migration.

### 2.5 Genomic Offset Validation Using Phenotypic Data

To provide an illustrative assessment of the modeled genomic offset for real‐world relevance, we used phenotypic data from two common garden trials in Canada. The first, located in Indian Head (Saskatchewan, long: −103.66, lat: 50.53) and established in 2014, includes five populations spanning a longitudinal gradient within the province (Figure S2). Each population is represented by 15 genotypes planted in triplicate. Annual growth increments from 2015 to 2019 were recorded. The second trial, established in Alberta in 2003 (long: −115.5, lat: 53.3), includes three populations from Alberta and one from Saskatchewan. Among the available traits evaluated, including survival rate, only the height of five‐year‐old trees (Saskatchewan trial) and the volume of 20‐year‐old trees, as it integrates both diameter and height (Alberta trial), were retained for model validation, as they are considered good indicators of long‐term performance. Considering that the tree age effect is important, as growth variability is high in juvenile trees, mean traits were calculated per population.

Following Lachmuth et al. (2023), we built linear models to assess the relationship between growth (height and volume) and the genomic offset for each common garden separately. While the populations used in the common gardens are not identical to those used in the genomic offset model, they originated from the same geographic regions. Employing the trained GF model, we predicted the genomic composition for the populations used in the common garden.

The raw genomic offset as well as the scaled genomic offset were then calculated and standardized using the climate data from the population's original location and its current location in the common garden, following the methods described earlier. For future climate projections, we used climate data corresponding to the years in which phenotypic data were collected: 2023 for the Alberta trial and 2019 for the Saskatchewan trial, respectively. Using these datasets, we fitted linear models with the population mean growth as the response variable and the standardized genomic offset as the fixed effect.

To explore an alternative approach, we fitted separate linear models with polyploidy as the response variable to test if a higher polyploidy correlated with the offset. We used range‐wide population data with available polyploidy information (Goessen et al. 2022, 2026; Goessen, Isabel, Wehenkel, et al. 2025). We calculated the proportion of polyploid individuals for each population and used it, expressed as a percentage, as the response variable in the models, following the same procedure as for the phenotypic data. For the offset, we employed the local offset values using the LFMM candidate SNPs and the SSP3‐7.0 scenario as described earlier. This analysis was motivated by the hypothesis that polyploidy may buffer maladaptation through increased genetic and physiological flexibility—a mechanism not captured by genomic offset models restricted to diploid individuals and therefore potentially leading to an underestimation of adaptive capacity.

## 3 Results

### 3.1 Gradient Forest Models

The Gradient forest model using the full SNP dataset selected 3192 SNPs (29% of all SNPs) with positive predictive power (Table 2). In comparison, the neutral dataset retained only 101 SNPs (26%), while the candidate SNP datasets consistently showed higher proportions of model‐selected loci: the LFMM dataset retained 316 SNPs, representing 75% of the original candidate SNPs, and the Bayenv2 dataset retained 138 SNPs (49%).

Substantial overlap was observed between the datasets. A large proportion of the LFMM and Bayenv2 candidate SNPs were also present in the full dataset, and 77 SNPs were shared between the two candidate SNP datasets.

Among all models, the LFMM candidate SNP model performed best, with a mean *R* ^2^ of 22%, indicating the highest explained genetic variation in relation to climate variables. The model based on neutral SNPs (18%) surprisingly outperformed the full dataset model (16%), while the Bayenv2 candidate model (17%) yielded similar explanatory power to the full dataset model.

Across all models, except the Bayenv2 candidate SNP dataset, the climate variables Eref (Hargreaves reference evaporation), SHM (Summer Heat‐Moisture Index), CMD (Climatic Moisture Deficit), MSP (Mean Summer Precipitation), and TD (Temperature Difference or Continentality) were consistently identified as the most important predictors of genomic variation. Notably, SHM in the LFMM candidate SNP model had the highest overall variable importance (*R* ^2^ 0.014; Table S2; Figure 2, Figure S3, Figure S5). SHM, which integrates the number of summer days above 5°C with precipitation, was associated with two distinct allele frequency turnovers, highlighting its ecological relevance. Eref showed the greatest importance in both the full and neutral datasets and was linked to a sharp allele frequency turnover around 1000 mm/year, indicating a strong genomic sensitivity to changes in evapotranspiration across those regions (Figure 3). Overall, moisture‐related variables had a greater influence on genetic variation than temperature‐related ones. We noted that in the Bayenv2 model, Moran's eigenvector values showed the highest *R* ^2^ values, suggesting that spatial autocorrelation played a stronger role in explaining genetic variation than climate alone (Figure 2).

**FIGURE 2:**
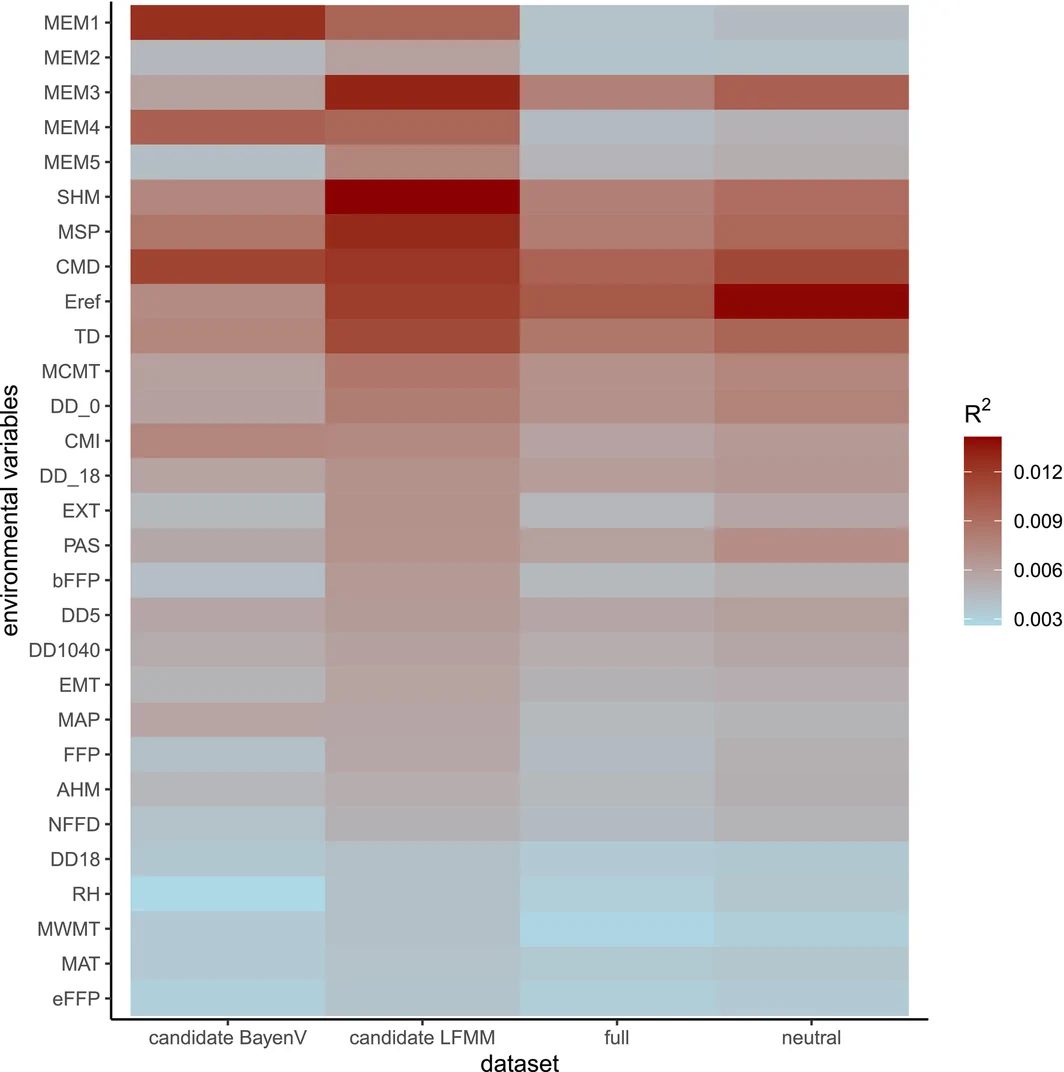
Variable importance of climate variables (SHM to eFFP) and Moran's eigenvector values (MEM1‐5) in the full, neutral, and candidate datasets. Climate variables can be found in Table 1. Sorted by variable importance in candidate LFMM dataset.

**FIGURE 3:**
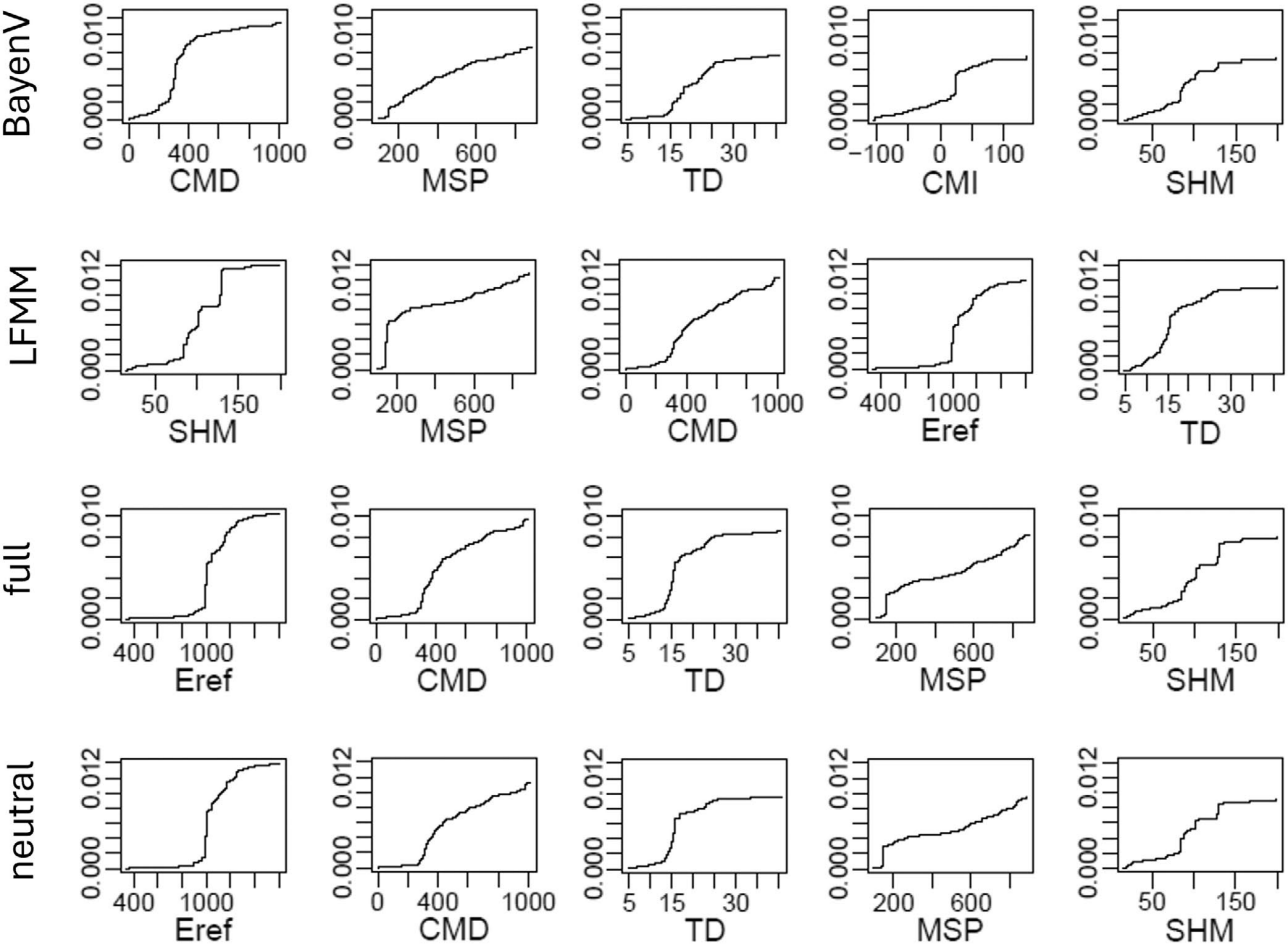
Turnover functions for the different climate variables per SNP dataset for the gradient forest model. Climate variables are found in Table S2.

The predicted genomic variation across the full distribution range of *P. tremuloides* revealed broadly similar spatial genetic patterns among datasets, with the neutral model differing the most from the others (Figures 4, S4). The species' southeastern range region appeared genetically differentiated from most of the distribution. Similarly, Mexican populations were clearly distinct from those in the northern range, with genetically unique regions also emerging in Alaska and the Rocky Mountains. Overall, the results revealed a pronounced north–south gradient in genomic structure, reflecting adaptation to varying climatic regimes across latitudes.

**FIGURE 4:**
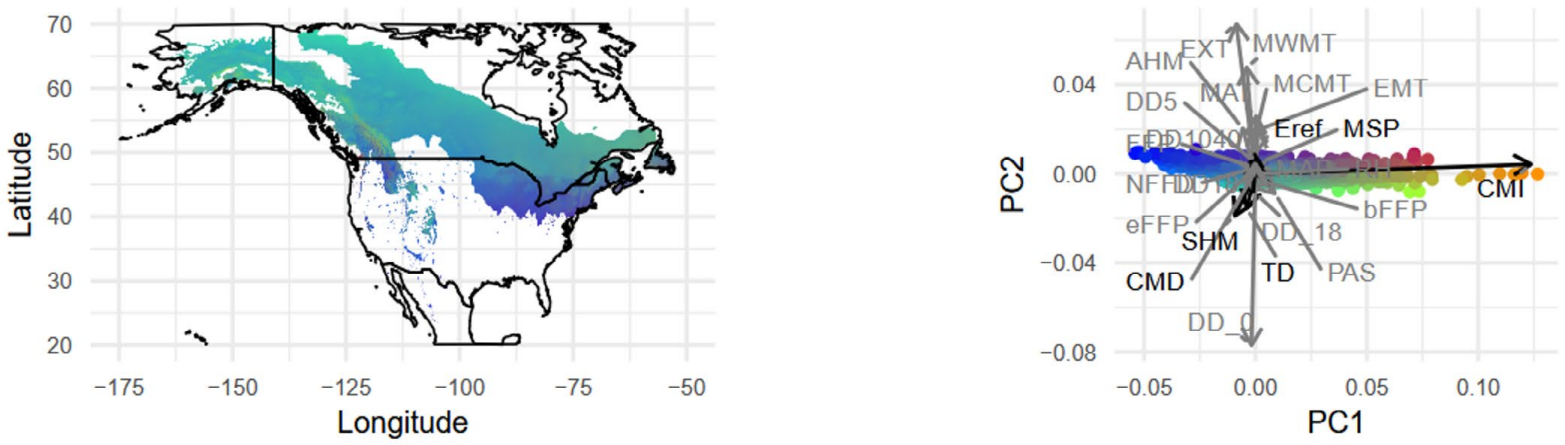
Predicted genomic variation modeled across *P. tremuloides* entire distribution range for the LFMM dataset. The first three principal components in a PCA using all climate variables were used to define an RGB color palette. Black arrows indicate selected climate variables used in further analysis. Similar colors indicate similar genomic composition among regions.

When comparing GF models run for the different genetic clusters, the model including the full species range showed the highest explanatory power (22%), followed by WUS and NWNA clusters (16% and 12%; Table S3). In contrast, models for the NENA and MX genetic clusters showed the lowest explanatory power (8% and 7%).

### 3.2 Genomic Offset

Genomic offset projections revealed clear spatial trends and climate scenario effects across the species range, highlighting regions at greatest risk for maladaptation under future conditions (Figure S5). Offset values increased with scenario severity, with SSP5‐8.5 producing the most widespread and intense high‐offset areas, followed by SSP3‐7.0 and SSP2‐4.5. The southern and western range margins, particularly in Mexico and the southwestern U.S.A., consistently indicated the highest offset values across all datasets and scenarios, suggesting a strong genomic mismatch under future climates. In contrast, populations in the northeast and boreal regions (e.g., eastern Canada and parts of the Great Lakes) showed lower offset values, indicating greater genomic‐climatic compatibility, although even these areas became more vulnerable under severe warming. Notably, localized regions in Alaska also exhibit persistently high offsets across scenarios and datasets, sharply contrasting with surrounding areas. Patterns were broadly consistent between the LFMM and Bayenv2 candidate datasets, although Bayenv2 showed slightly more fragmented high‐offset zones. The full and neutral datasets predicted lower and more continuous offset values, except for the neutral dataset under SSP5‐8.5, which showed extensively high offsets across nearly the entire range.

These results underscore that both dataset choice and climate scenario severity substantially influence offset predictions. Nevertheless, all models converge in identifying southern and western populations as facing the highest genomic offset, and therefore, the greatest risk of maladaptation under future climate change.

### 3.3 Recipient and Donor Importance

Given that the LFMM candidate model explained genomic variation most effectively, we used it to calculate donor and recipient importance. The resulting maps (Figure 5) showed pronounced spatial variation in the species' adaptive potential across ecozones.

**FIGURE 5:**
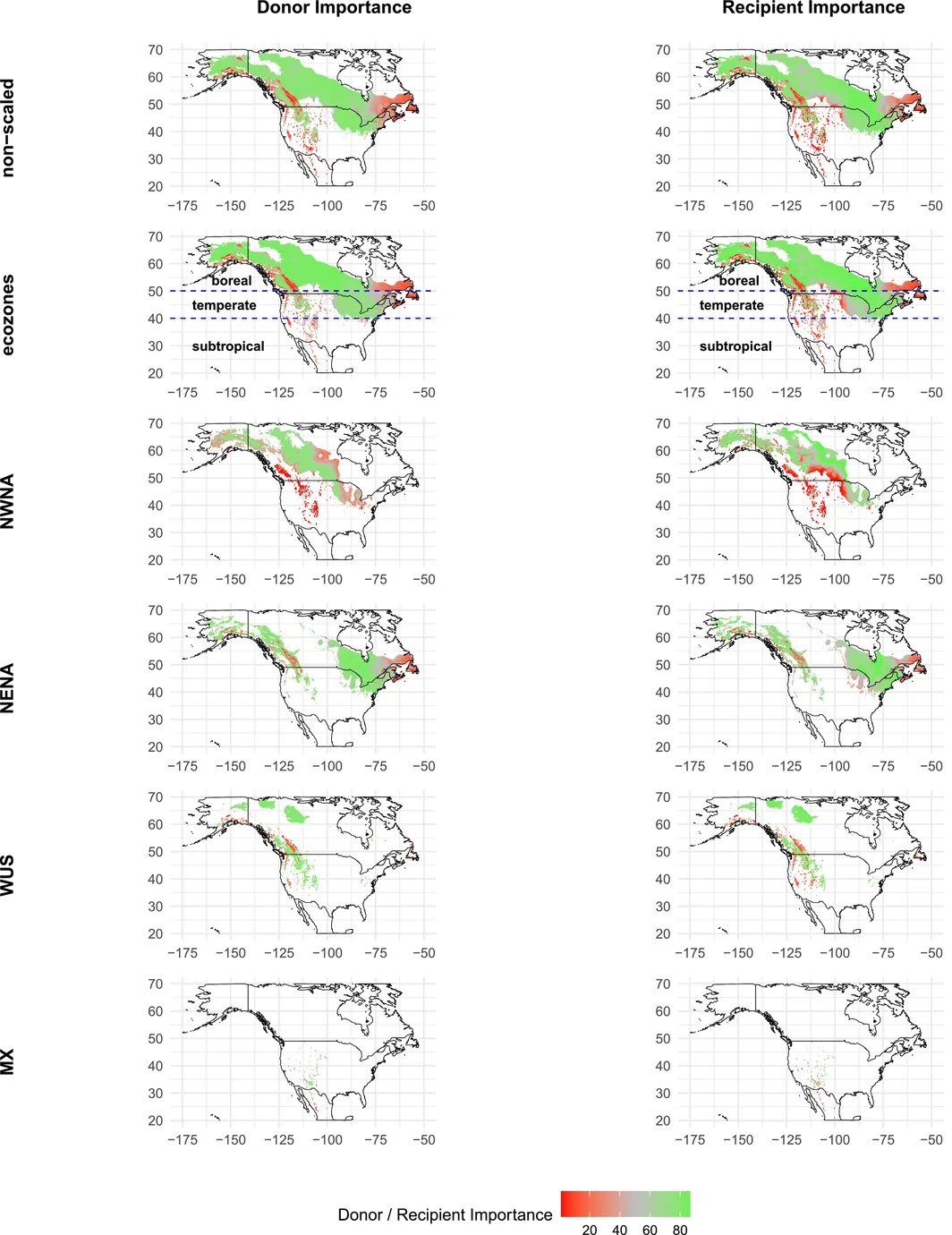
LFMM dataset for evaluating populations' donor/recipient importance on full range, or calculated only in ecozones or scaled on each genetic cluster (NWNA, NENA, WUS, and MX) under climate scenario SSP3‐7.0.

In the Boreal zone, donor and recipient importance were consistently high (median: 82.2% and 82%), particularly across central Canada, suggesting this region may be key for future adaptation by providing sources of adaptive alleles and recipients under future climates (Table S4). In contrast, the range's eastern edge exhibits low importance values, indicating reduced suitability and limited potential as future donors or recipients. In the Temperate zone, recipient importance was lower overall (66.6%), especially in the western areas, which appeared increasingly unsuitable under future climates. Donor importance was also more fragmented in this region (median: 68.8%), with a west‐to‐east decline, suggesting that western temperate populations may have limited broad‐scale adaptive potential. In the Subtropical zone, both donor and recipient importance were lowest (37.5% and 38.1%), although pockets of moderate donor potential existed in northern Mexico, indicating localized contributions of adaptive alleles.

When examining genetic cluster‐specific, scaled genomic offsets, distinct intra‐cluster spatial patterns emerged. These patterns were often more detailed than those derived from range‐wide estimates, emphasizing the value of regional analysis (Figure 5). In the NWNA, donor and recipient importance peaked in central Canada (max: 78.6% and 90.3%) and dropped sharply in the southern part (min: 0%) (Table S4). In the NENA, eastern Canada hosted high donor importance (max: 80.7%), while recipient importance was highest in the central part of the cluster but very low in the far east, especially in Nova Scotia and Newfoundland, where both metrics approach zero. The WUS cluster exhibits consistently high donor and recipient importance (medians: 79.1% and 82.4%), especially among U.S.A. populations, with low variability, suggesting uniform within‐cluster compatibility and strong potential for local gene flow. In contrast, the MX cluster showed the greatest variability, with importance values ranging from 1% to 88%, reflecting the ecological and genetic uniqueness of these southernmost populations.

Comparing ecozone‐ and cluster‐based scaling to range‐wide calculations revealed a finer resolution in adaptive relationships. These localized approaches identified more suitable donor populations, particularly in regions like the subtropical ecozone, which appeared less important in global‐scale analyses. The most striking example was the WUS cluster, which showed much higher donor and recipient importance when standardized within the cluster. In general, southern clusters exhibited increased importance under local scaling, while northern clusters (i.e., NWNA) showed more regions with future unsuitability.

### 3.4 Genomic Offset Validation

The linear models evaluating the relationship between the genomic offset and fitness (e.g., growth) provided limited statistical support for validating the genomic offset predictions, as evidenced by consistently high *p*‐values (*p* > 0.05) and very low *R* ^2^ values (< 0.01; Table S5). This limited explanatory power likely reflects both the high phenotypic variation within populations and the small number of populations included in the analysis (Figure S6A). Overall, models based on raw and scaled offsets produced similar results.

The linear model including local offset and polyploidy had low (*R* ^2^ = 0.09) yet significant explanatory power (Table S5). The model's positive slope suggests higher offset is associated with higher polyploidy (Figure S6B).

## 4 Discussion

This study presents a framework to assess the climate‐related risk of maladaptation in *P. tremuloides* by addressing two key questions: how to calculate and standardize the genomic offset for a widely distributed species, and how to identify both at‐risk and potentially resilient populations under future climates. By leveraging a broad genomic dataset covering the species' full range and applying a standardized offset method adopted from Lachmuth et al. (2023), we identified spatial patterns in vulnerability and candidate source populations for assisted migration.

### 4.1 Selective Pressure of Climate Variables and Dataset Effects on Genomic Offset Predictions

Moisture‐related variables such as the Hargreaves reference evaporation (Eref), the Summer Heat‐Moisture Index (SHM), and Climatic Moisture Deficit (CMD) consistently emerged as the strongest genetic variation predictors in *P. tremuloides* , surpassing temperature‐based variables. This pattern reflects the species' riparian ecology, where moisture availability strongly influences adaptive genetic differentiation. For example, spring phenology in aspen is closely linked to precipitation regimes, with dry winters and springs negatively correlated with growth (Li et al. 2010). SHM and mean summer precipitation were especially influential, consistent with findings from the western U.S.A., where aridity and seasonal precipitation ratios best predicted aspen presence (Rehfeldt et al. 2009). Environmental aridity also correlates with higher polyploidy frequencies (Goessen et al. 2022), but triploids are also reported to show higher drought vulnerability in aspen (Greer et al. 2018; Blonder et al. 2021). Similar moisture‐driven genomic signals have been observed in *P. balsamifera* , where offsets were most strongly associated with winter and summer precipitation (Gougherty et al. 2021).

CMD was among the most influential variables in the dataset, while vapor pressure deficit (VPD) showed weaker effects, despite being a recognized growth sensitivity driver (Mirabel et al. 2023). This apparent mismatch suggests that traits with weaker phenotypic buffering may impose stronger selective pressure, whereas traits with robust plastic responses—such as growth linked to freeze days or FFD; (Girardin et al. 2022; Mirabel et al. 2022)—may reduce the need for genetic differentiation. Thus, strong phenotypic plasticity could dampen climate‐driven selection and facilitate broader resilience. Moisture variable predominance conflicts with broader evidence that drought adaptation is harder to detect in trees than temperature adaptation due to its fine spatial and temporal variability, polygenic basis, and interaction with soils and topography (Candido‐Ribeiro and Aitken 2024).

These environment‐genomic associations were consistent across SNP datasets, despite differences in marker selection. Although the neutral dataset included only intergenic SNPs, the variable importance patterns were similar to those of the candidate SNP datasets. The full dataset showed the lowest predictive power, likely due to the inclusion of many non‐informative loci. GF models nevertheless converged on largely overlapping subsets of informative SNPs across datasets, underscoring the robustness of the signal. Candidate SNP datasets (Bayenv2 and LFMM) differed in performance, with LFMM explaining more genetic variation, while Bayenv2 showed stronger spatial autocorrelation. Yet, overall explained variance remained similar across datasets, a pattern also observed in other studies (Fitzpatrick et al. 2021; Lachmuth et al. 2024; Lind et al. 2024). This may reflect that isolation‐by‐environment accounts for only a small total genomic variation fraction (Lind and Lotterhos 2024), and that the offset functions more as a predictor for polygenic adaptation, which can be approximated even with random SNPs if population structure is not fully accounted for (Gain et al. 2023; Lotterhos 2024; Rellstab and Keller 2025).

Spatial predictions further illustrate these dynamics: southeastern populations appeared genetically distinct, likely reflecting the east‐west precipitation gradient, and Rocky Mountain populations showed localized genomic signatures tied to topographic heterogeneity. A pronounced north‐south gradient also emerged, potentially driven by extensive longitudinal gene flow via wind‐dispersed pollen (Gillet 2017; Van de Water et al. 2007).

### 4.2 Genomic Offset Across a Wide Distribution Range

Although offset values varied among the differently scaled datasets and climate scenarios, consistently high‐offset regions emerged. Populations in Mexico and the southern U.S.A. displayed the highest offsets, indicating heightened vulnerability to climate‐driven maladaptation. This pattern is notable because Mexican populations are often considered more drought‐adapted and thus potentially better suited to future conditions. Their consistently high offsets, however, suggest that local adaptations may not align with the direction or magnitude of projected environmental change. Isolated Alaskan populations also showed high offsets, likely reflecting poor sampling coverage and reduced model accuracy. By contrast, northern populations exhibited the lowest offsets, consistent with expectations that tree ranges will shift northward with rising minimum temperatures, while southern populations are projected to decline (Boisvert‐Marsh and De Blois 2021; Desprez et al. 2014; Lima et al. 2024; McKenney et al. 2007). Still, the sensitivity of growth in aspen to winter temperature could limit its northwards expansion (Girardin et al. 2022).

Polyploidy and clonality, particularly common in the southwestern range, may help buffer maladaptation by enhancing genetic and physiological flexibility and sustaining locally adapted genotypes under environmental stress but were not captured by genomic offset models. Triploidy is most frequent in drought‐prone western U.S.A. populations (Goessen et al. 2022; Mock et al. 2012), likely reflecting selection pressures favoring polyploidy (Mock et al. 2012). Whole‐genome duplication can enhance adaptive potential by increasing genetic variation, altering gene expression, and modifying physiological traits such as transpiration and photosynthesis (Van De Peer et al. 2017, 2021). Because our offset calculations were restricted to diploid individuals, they may underestimate the adaptive capacity of polyploid individuals. The positive correlation between polyploidy and the offset observed in these linear models suggest a potential adaptive role that warrants further investigation with broader datasets.

Southwestern populations, while showing high offsets and reduced genetic diversity, may nonetheless hold disproportionate evolutionary potential. Callahan et al. (2013) describe them as historically stable‐edge populations, with low within‐population diversity, strong geographic structure, and limited evidence of northward expansion. Such traits imply vulnerability under climate change, yet these same populations may carry unique adaptive variation of high conservation value. The complex topography of the region, with multiple mountain ranges and basins, likely facilitated elevational shifts during glacial‐interglacial cycles, supporting long‐term persistence and local adaptation. Thus, despite genomic signals of vulnerability, these populations may harbor important adaptive traits. Consequently, high offset values do not equal low conservation value, which is particularly true for rear‐edge or stable‐edge populations.

The offset patterns also reflect broader climatic structuring across the range. Distinct clusters correspond to the semi‐arid western U.S.A. versus the mesic, continental climates of Canada and Alaska (Callahan et al. 2013; Mock et al. 2012). Southern clusters consistently exhibited higher offsets, consistent with their isolation and lower genetic diversity. Especially in the typically small and isolated, southernmost populations (in Mexico), an extreme excess of heterozygosity was found in genotype‐poor populations with common asexual reproduction counteracting the inbreeding. Therefore, asexual reproduction may play a contributing role in the ability of the Mexican populations to persist and adapt to extreme ecological conditions (Hernández‐Velasco et al. 2025). Microsatellite analyses confirm lower allelic richness in southwestern populations (Callahan et al. 2013), which may contribute to their elevated offsets. Western genotypes also show reduced germination under drought compared to eastern ones (Goessen et al. 2022), suggesting a drought‐avoidance strategy that suppresses germination during unfavorable conditions. While this may buffer short‐term stress, widespread dieback events, such as in 2002, illustrate the continued ecological vulnerability of these populations, with suitable habitats predicted to shift upward in elevation in the Rocky Mountains (Rehfeldt et al. 2009).

Scaling donor and recipient importance within clusters provided finer resolution than range‐wide estimates. For example, WUS cluster‐specific analyses revealed higher suitability of both donor and recipient populations than suggested by the global models, which may overestimate maladaptation by ignoring local adaptation. Given the broad geographic and climatic range of aspen, large‐scale translocations, such as moving genotypes from Mexico to Canada, are unlikely to be ecologically feasible. Soil microbiome communities or locally adapted phenological traits, including bud break requirements, are shaped by regional climates and may not function under mismatched conditions (Li et al. 2010; McKown et al. 2018; Fijarczyk et al. 2025). Ecozone‐ and cluster‐based standardization therefore provides a more ecologically realistic framework for assessing the genomic offset. By aligning analyses with ecological classification and genetic structure, these approaches improve the resolution of intra‐regional adaptive patterns, help identify locally suitable donor and recipient populations, and offer more practical guidance for regionally adapted conservation and assisted gene flow strategies.

### 4.3 Limits and Applications of Genomic Offset for Assisted Migration

Genomic offset models, such as Gradient forest, capture only genetic and environmental correlations and therefore provide an incomplete representation of adaptive potential (Láruson et al. 2022). Key biological processes, including reproductive strategies, hybridization, phenotypic plasticity, polyploidy, the Meselson effect, and epigenetic modifications, are not accounted for. For example, hybridization has been shown to play an important role in *Populus* evolution: introgression can introduce novel alleles and facilitate rapid adaptation to changing environments (Lexer et al. 2004; Suarez‐Gonzalez et al. 2018). Examples include *P. balsamifera* alleles introgressing into *P. trichocarpa* , which have been shown to underlie adaptively significant traits, including an extended range and clinal adaptation (Suarez‐Gonzalez et al. 2018). Recent evidence showed that introgression between *P. fremontii* and *P. angustifolia* enhanced survival under warmer, drier climates (Hord et al. 2025). In addition, a review by Suarez‐Gonzalez et al. (2018) described adaptive introgression broadly across plant species and specifically highlighted *Populus* as a key case where gene flow contributes to adaptive variation along environmental gradients.

Similarly, soil type, disturbance, and successional dynamics further shape habitat suitability (Rehfeldt et al. 2009). Even belowground interactions differed for trees among genetic clusters of *P. tremuloides* . A recent study showed that host genotype and soil properties jointly shape microbial community composition, in which Mexican populations supported soil microbiomes enriched in nitrogen‐fixing bacteria, while boreal populations showed stronger associations with ectomycorrhizial fungi—consistent with the functional divergence of mutualism linked to contrasting environmental conditions (Fijarczyk et al. 2025).

At the same time, trees may be capable of adapting faster than previously assumed. Kremer et al. (2025) showed that adaptive evolution can occur within a few generations, with microevolutionary responses closely tracking climate change. Large effective population sizes, strong spatial genetic structure, and the interplay between plasticity and genetic variation enhance resilience and can accelerate adaptive responses, suggesting a greater evolutionary potential than the genomic offset approach alone implies (Kremer et al. 2025). On the other hand, current climate change may not be represented in past climates and thus may not have sufficient standing adaptive genetic variation. Forest disturbances such as drought‐induced mortality and wildfire will nevertheless increase the demand for reforestation material, raising the need for robust guidelines to minimize future maladaptation (Palik et al. 2022). Genomic offset estimates provide one starting point but must be applied cautiously when informing assisted migration. Genomic offset calculation prediction accuracy breaks down under increasingly novel environments (Rellstab and Keller 2025). For this reason, we restricted predictions to the current distribution range, since out‐of‐range translocations effectively introduce a new species to an ecosystem rather than new genotypes within a species (Peterson St‐Laurent et al. 2018). Further, introducing maladapted genotypes risks outbreeding depression or the disruption of local interactions, although high gene flow in wind‐pollinated species like aspen makes such risks relatively rare and predictable (Aitken and Whitlock 2013; Frankham et al. 2017).

Given aspen's wide distribution and strong genetic structure, fine‐scale approaches are particularly valuable. Calculating donor and recipient importance within ecozones or genetic clusters provides higher resolution and reduces mismatching regional adaptations. Moreover, no single seed source is consistently superior; pooling multiple seed sources has been shown to enhance genetic diversity and adaptive potential (Prakash et al. 2024). Such strategies can help balance local adaptation with the capacity to respond to uncertain future conditions. Resilient forest ecosystems require more than just genetics; for example, more diverse forests lead to greater resilience (Peterson St‐Laurent et al. 2018).

Finally, reliable validation of genomic offset estimates requires robust phenotypic data. For example, evidence from an Alberta common garden trial indicated that transferring aspen populations to areas where extreme minimum temperatures are not more than 4°C cooler than their origin is generally safe for reforestation, corresponding to a northward movement of approximately 5°–10° in latitude (Ding and Brouard 2022). These trials also revealed a trade‐off between cold adaptation and growth, with height growth genetically correlated with the duration of the growing season. These linear models included only a small subset of populations, limiting validation over different population ranges. Range‐wide common garden and provenance trials are therefore essential to test predictions and develop evidence‐based recommendations. Comparisons of climatically contrasting populations, such as those from Mexico and Alaska, would be particularly valuable. While such resources are well established for conifers like white spruce or jack pine (Pedlar and McKenney 2017; Risk et al. 2021), they remain scarce for broadleaf species like aspen, underscoring a critical gap for future research. Given the limited number of populations and restricted geographic scope, our phenotypic validation should be interpreted as illustrative rather than confirmatory evidence of genomic offset performance.

## 5 Conclusions

This study demonstrates that the genomic offset is a powerful tool to identify regions with aspen populations that may be at risk for climate‐driven maladaptation, but it also highlights its limitations, as key biological and ecological processes influencing adaptive capacity are not captured. Choosing the SNP dataset had only minor effects on model outcomes, suggesting that identifying candidate SNPs prior to running GF models may be unnecessary. Consistently, southern and western range margins, particularly Mexico and the southwestern U.S.A., exhibited the highest offsets, indicating elevated vulnerability despite their apparent drought adaptations, whereas northern populations exhibited the lowest offsets. Nevertheless, high offsets do not imply low conservation value, as rear‐edge populations may retain unique adaptive variation and buffering mechanisms not captured by genomic offset estimates. Applying fine‐scale approaches, such as ecozone‐ or cluster‐based scaling, enhanced resolution and revealed locally relevant donor and recipient populations, providing a basis for regionally tailored assisted gene flow strategies. To translate these genomic predictions into reliable management recommendations, integration with range‐wide phenotypic data from common garden and provenance trials is essential to guide conservation and ensure the long‐term resilience of aspen. Table 3 provides the definitions for certain terms relevant to this study.

**TABLE 3:** Nomenclature.

assisted gene flow	intentional movement of individuals or genetic material within a species' range to enhance the adaptive potential of populations facing environmental change
assisted migration	intentional relocation of species or populations to areas outside their current range to help them survive and adapt to changing environmental conditions
clonemate	individual that originates from the same genetic clone as another, sharing an identical genotype produced through asexual reproduction
donor importance	recipient locations proportion where a given donor population could be transferred without exceeding the maladaptation threshold
ecological regions	land or water areas with similar climate, geology, soils, physiography, and the resulting distinctive natural plant and animal communities
genomic offset	genetic mismatch between current allele frequencies and those predicted under future climate scenarios
genomic vulnerability	insufficiency of a population's existing genetic variation to maintain fitness under future climate scenarios, reflecting limitations in adaptation
phenotypic buffering	compensatory process that stabilizes an organism's phenotypic traits against variations in its genes and/or the environment
plasticity	an organism's adaptability to changes in its environment or differences between its various habitats
ploidy	number of chromosome sets in a cell and/or in the cells of an organism
recipient importance	donor cells (= donor populations) proportion able to immigrate without exceeding the maladaptation threshold
risk of maladaptation	likelihood that the current genetic composition will be mismatched to future environmental conditions, resulting in reduced fitness, growth, or survival
stable‐edge population	scenario where population(s) persist through climate oscillations, typically in areas with varied topography that facilitate elevational shifts to match suitable climatic profiles over time (Callahan et al. 2013)

## Funding

This research was funded by FRQNT‐CONACYT‐FRQ‐2016: 279459 “Genome‐wide scans for detecting adaptation to climate and soil in *Populus tremuloides* as the most widely distributed tree species in North America” (genotyping and sampling) to I.P. and C.W., NSERC Discovery grant RGPIN/04748‐2017 (SNP calling against the new reference genome; sampling in Texas) to I.P. and a postdoctoral fellowship from the FRQNT bourses des recherche postdoctorale (B3X: 2024‐2025: 348498) program to M.Z. Additional funds were provided through Genome Québec's Genomics Integration Program grants to I.P. and N.I. (“Genomics‐Informed Forest Certification and Wood Traceability Tools and Protocols” 2022–2025).

## Conflicts of Interest

The authors declare no conflicts of interest.

## Supporting information


**Data S1:** eva70332‐sup‐0001‐Supinfo1.csv.

**Data S2:** eva70332‐sup‐0002‐Supinfo2.csv.

**Table S1:** Genetic cluster characteristics.
**Table S2:** Importance values for environmental variables for the different datasets.
**Table S3:** SNPs selected to build the GF models for the different genetic cluster.
**Table S4:** Donor and recipient importance for LFMM dataset with climate scenario SSP3‐7.0.
**Table S5:** The liner model parameters for assessing the relationship between genomic offset and fitness (growth) or polyploidy.

**Figure S1:** PCA of four genetic cluster (NWNA, NENA, WUS, and MX) using the full SNP dataset.

**Figure S2:** Source population locations of the common garden in Alberta and Saskatchewan trials (blue triangles), Saskatchewan trial (red circle), and for the Alberta trial (green circle). *P. tremuloides* ' distribution range in brown.

**Figure S3:** Scatterplots of variable importance of different datasets.

**Figure S4:** Predicted genomic variation modeled across *P. tremuloides* entire distribution range for the four tested datasets. The first three principal components in a PCA using all climate variables were used to define an RGB color palette. Black arrows indicate selected climate variables used in further analysis. Similar colors indicate similar genomic composition among regions.

**Figure S5:** Genomic offset (non‐scaled) for BayenV, LFMM, full, and neutral datasets with three different climate scenarios warmer colors (redder) indicate areas of higher predicted genomic offset.

**Figure S6:** Linear models representing the relationship between (A) offset and growth for the common garden trials in Alberta (red) and Saskatchewan (blue), and (B) offset and polyploidy.

## Data Availability

R scripts used in this study are openly available on Zenodo (DOI: 10.5281/zenodo.17434780). The SNP genotype datasets and phenotypic data from common garden trials are available upon request.
