# Supplementary material for: Assessing Future Maladaptation Risk in the Keystone Species Populus tremuloides
Source: Evol Appl. 2026 Oct 13;19(10):e70332. doi: 10.1111/eva.70332 (PMC13659280; doi:10.1111/eva.70332)
Supplement: Supplementary file 1 [file EVA-19-e70332-s001.pdf]

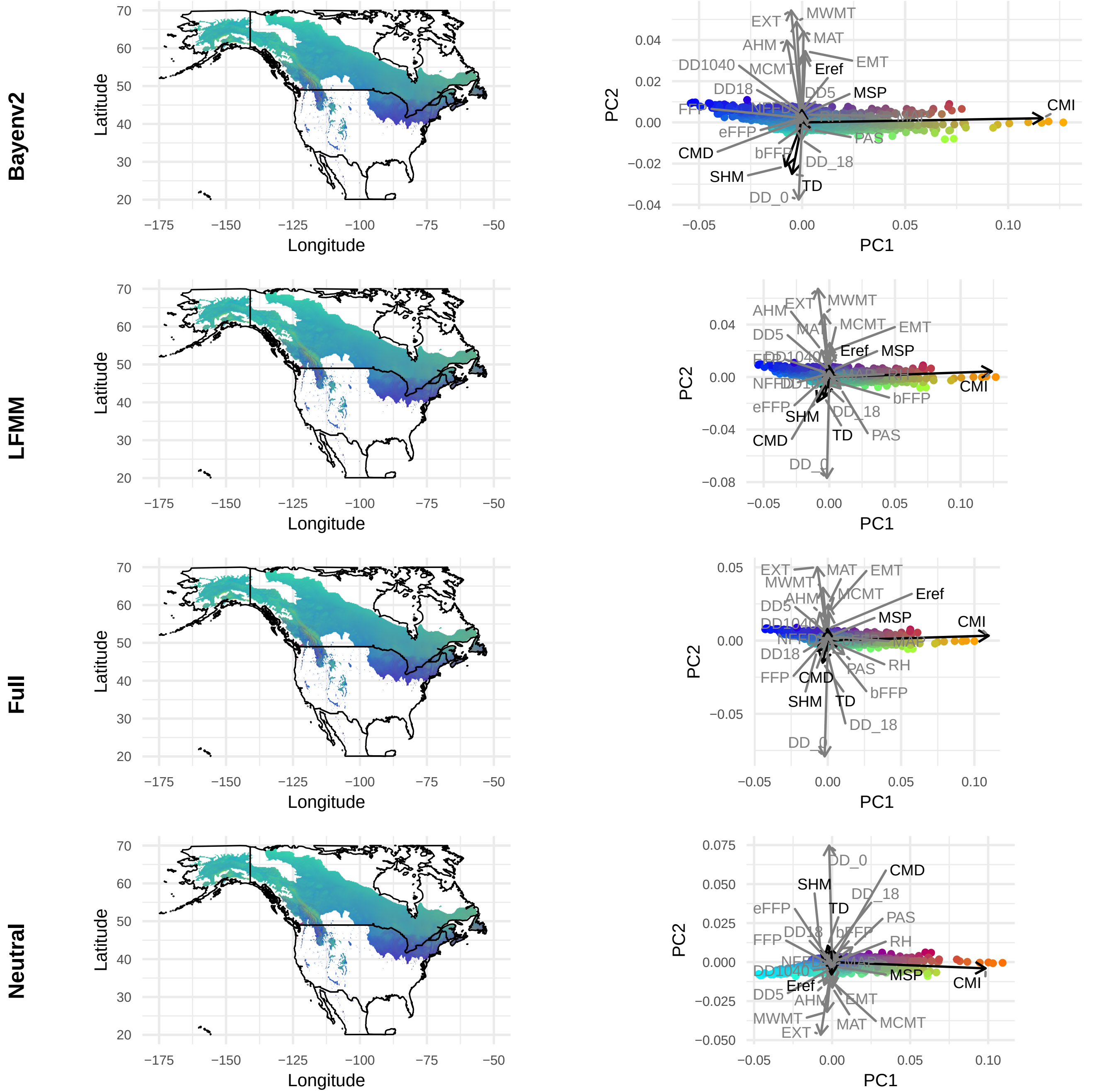

Bayenv2
LFMM
Full
Neutral
Latitude
Longitude
70
60
50
40
30
20
−175
−150
−125
−100
−75
−50
PC1
PC2
MWMT
EXT
MAT
AHM
EMT
DD1040
MCMT
Eref
DD18
DD5
MSP
FFP
NFFD
CMI
eFFP
PAS
CMD
bFFP
DD_18
SHM
TD
DD_0
RH
MAP
