# Supplementary material for: Assessing Future Maladaptation Risk in the Keystone Species Populus tremuloides
Source: Evol Appl. 2026 Oct 13;19(10):e70332. doi: 10.1111/eva.70332 (PMC13659280; doi:10.1111/eva.70332)
Supplement: Supplementary file 2 [file EVA-19-e70332-s002.pdf]

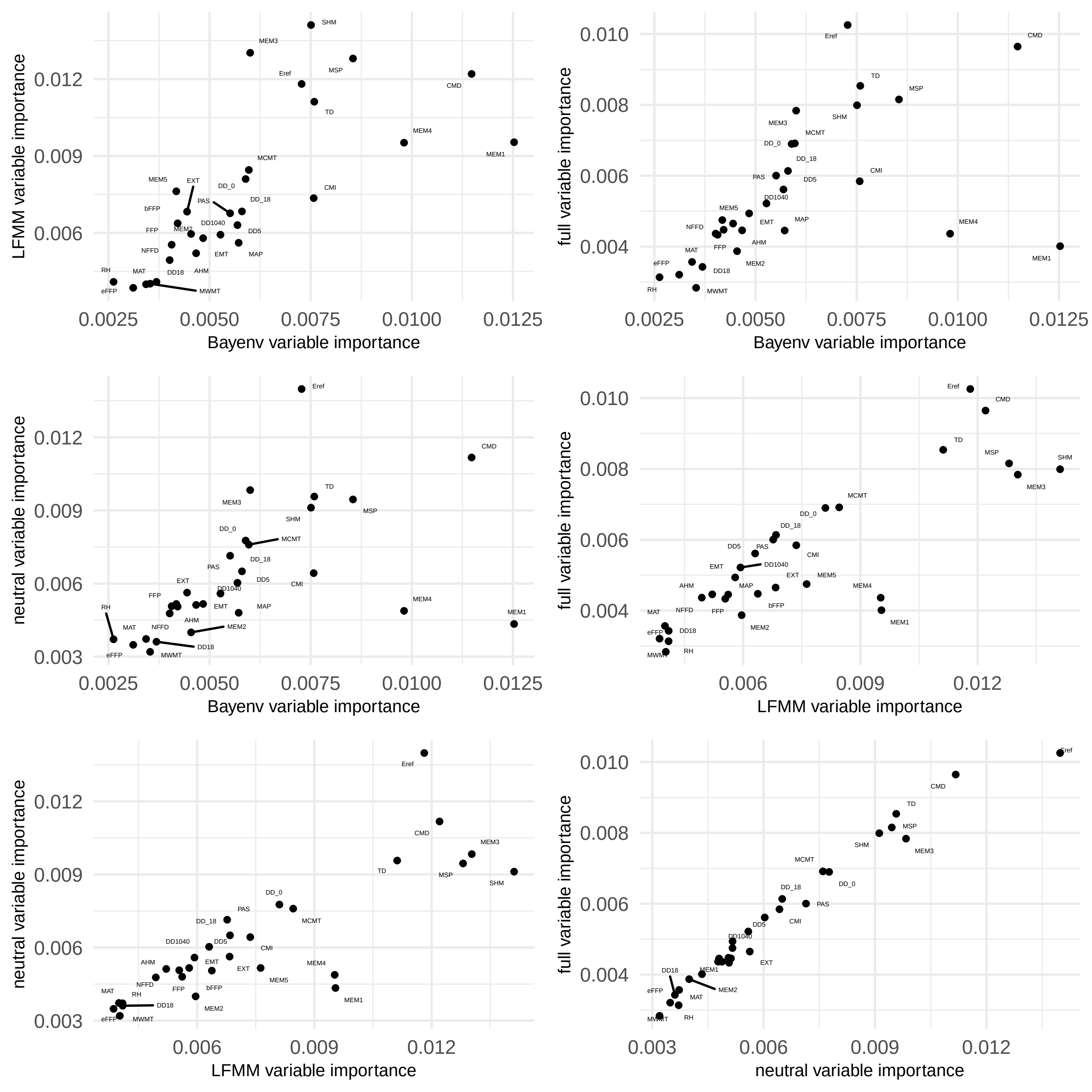

LFMM variable importance
0.012
0.009
0.006
SHM
MEM3
MSP
Eref
CMD
TD
MEM4
MEM1
MCMT
MEM5
EXT
DD_0
CMI
PAS
DD_18
bFFP
DD1040
FFP
MEM2
DD5
NFFD
EMT
MAP
RH
MAT
DD18
AHM
eFFP
MWMT
0.0025
0.0050
0.0075
0.0100
0.0125
Bayenv variable importance
full variable importance
0.010
0.008
0.006
0.004
Eref
CMD
TD
MSP
SHM
MEM3
MCMT
DD_0
DD_18
CMI
PAS
DD5
DD1040
MEM5
NFFD
EMT
MAP
MEM4
FFP
AHM
MAT
MEM1
eFFP
MEM2
DD18
RH
MWMT
0.0025
0.0050
0.0075
0.0100
0.0125
Bayenv variable importance
neutral variable importance
0.012
0.009
0.006
0.003
Eref
CMD
TD
MEM3
MSP
SHM
DD_0
MCMT
DD_18
PAS
DD5
CMI
EXT
FFP
DD1040
EMT
MAP
MEM4
MEM1
RH
AHM
MAT
NFFD
MEM2
DD18
eFFP
MWMT
0.0025
0.0050
0.0075
0.0100
0.0125
Bayenv variable importance
full variable importance
0.010
0.008
0.006
0.004
Eref
CMD
TD
MSP
SHM
MEM3
MCMT
DD_0
DD_18
DD5
PAS
CMI
EMT
DD1040
EXT
MEM5
AHM
MAP
MEM4
bFFP
MAT
NFFD
FFP
MEM1
eFFP
DD18
MEM2
MWMT
RH
0.006
0.009
0.012
LFMM variable importance
neutral variable importance
0.012
0.009
0.006
0.003
Eref
CMD
MEM3
TD
MSP
SHM
DD_0
PAS
MCMT
DD_18
DD1040
DD5
CMI
AHM
EMT
EXT
MEM4
MEM5
NFFD
FFP
bFFP
MAT
RH
DD18
MEM1
MEM2
eFFP
MWMT
0.006
0.009
0.012
LFMM variable importance
full variable importance
0.010
0.008
0.006
0.004
Eref
CMD
TD
MSP
SHM
MEM3
MCMT
DD_0
DD_18
PAS
DD5
CMI
DD1040
EXT
DD18
MEM1
eFFP
MAT
MEM2
RH
MWMT
0.003
0.006
0.009
0.012
neutral variable importance
