# Supplementary material for: Assessing Future Maladaptation Risk in the Keystone Species Populus tremuloides
Source: Evol Appl. 2026 Oct 13;19(10):e70332. doi: 10.1111/eva.70332 (PMC13659280; doi:10.1111/eva.70332)
Supplement: Supplementary file 3 [file EVA-19-e70332-s005.pdf]

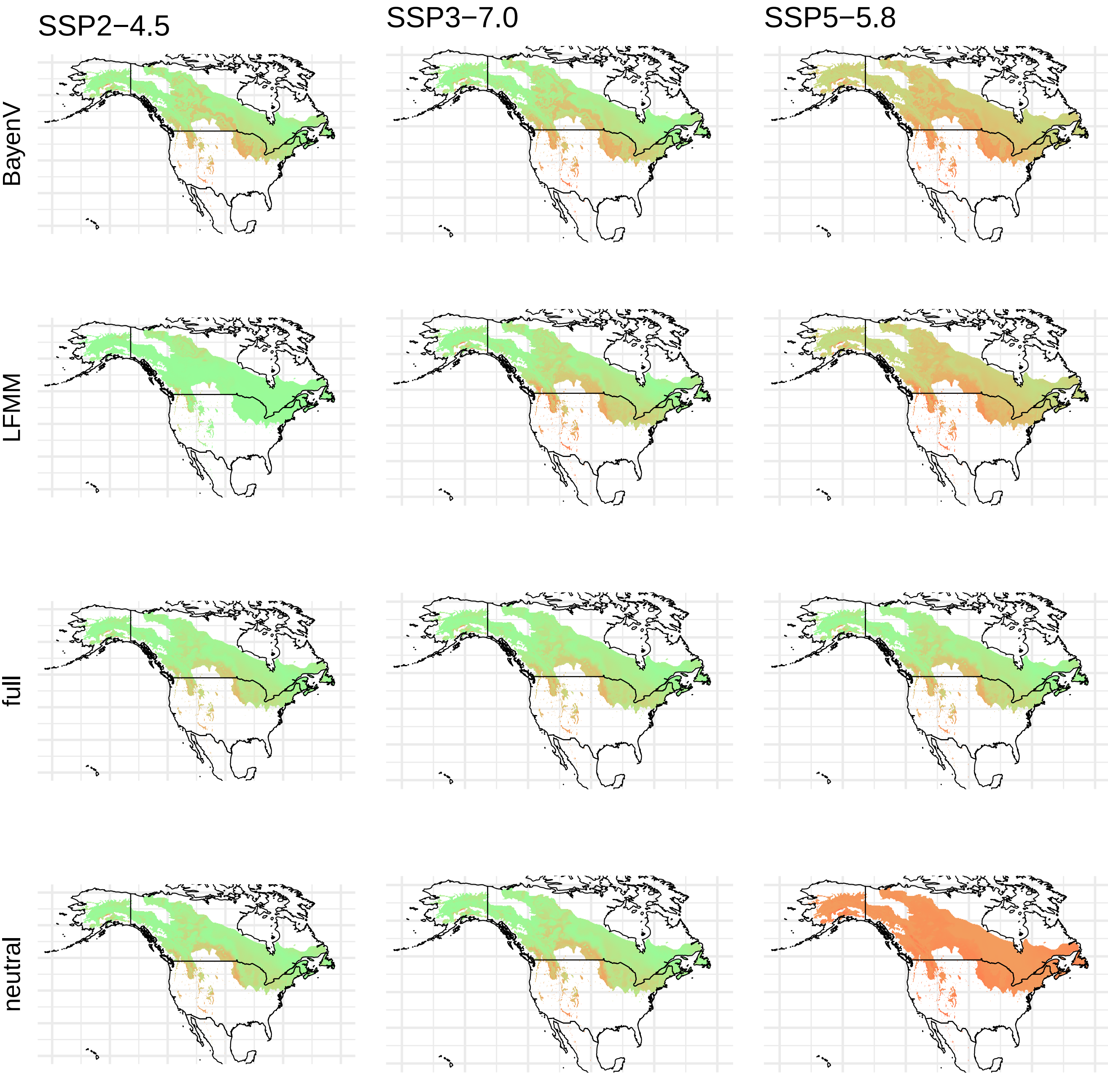

SSP2−4.5
SSP3−7.0
SSP5−5.8
BayenV
LFMM
full
neutral
