# Supplementary material for: Assessing Future Maladaptation Risk in the Keystone Species Populus tremuloides
Source: Evol Appl. 2026 Oct 13;19(10):e70332. doi: 10.1111/eva.70332 (PMC13659280; doi:10.1111/eva.70332)
Supplement: Supplementary file 4 [file EVA-19-e70332-s006.pdf]

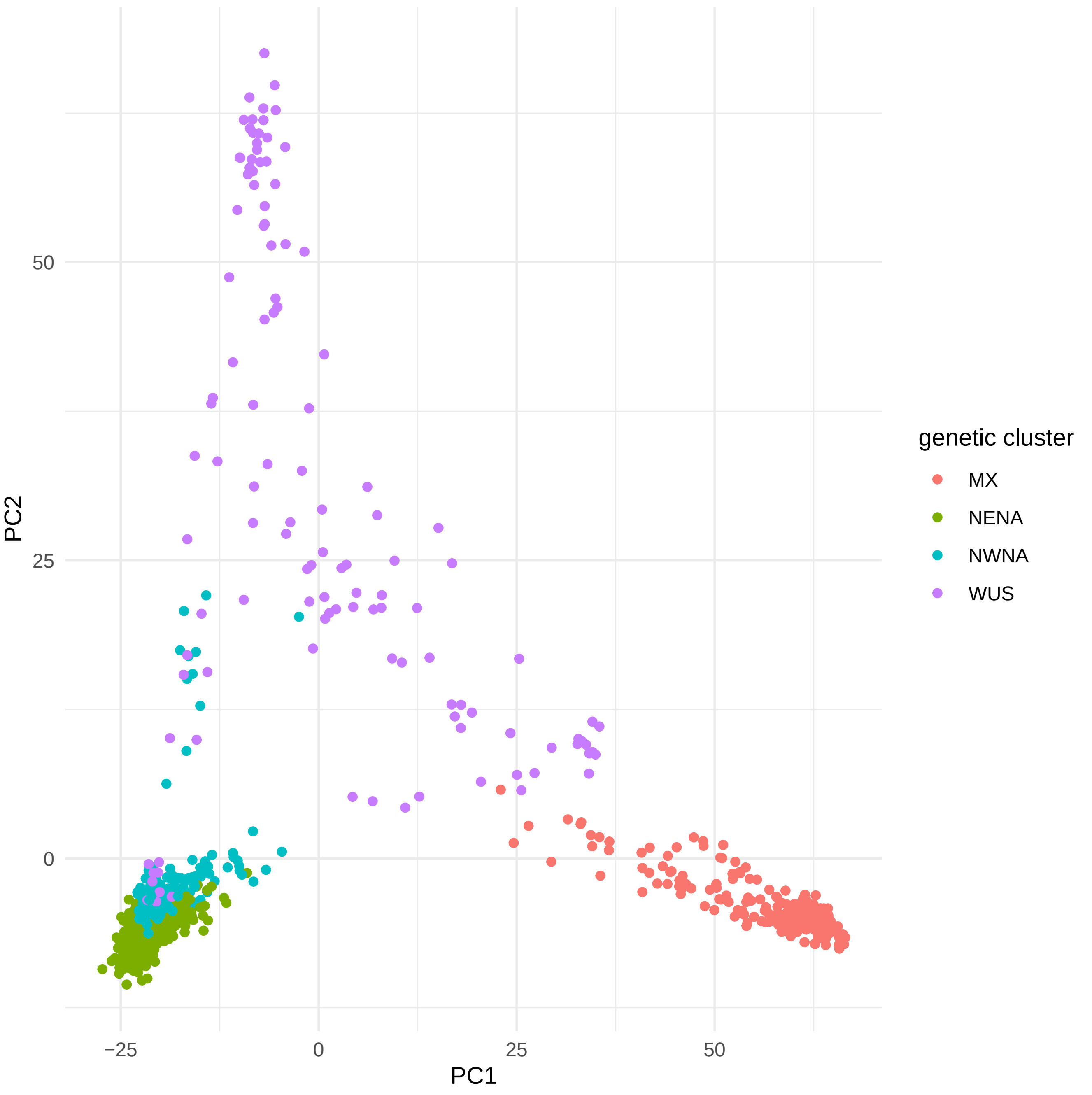

50
25
0
−25
0
25
50
PC1
PC2
genetic cluster
MX
NENA
NWNA
WUS
