# Supplementary material for: Assessing Future Maladaptation Risk in the Keystone Species Populus tremuloides
Source: Evol Appl. 2026 Oct 13;19(10):e70332. doi: 10.1111/eva.70332 (PMC13659280; doi:10.1111/eva.70332)
Supplement: Supplementary file 5 [file EVA-19-e70332-s007.pdf]

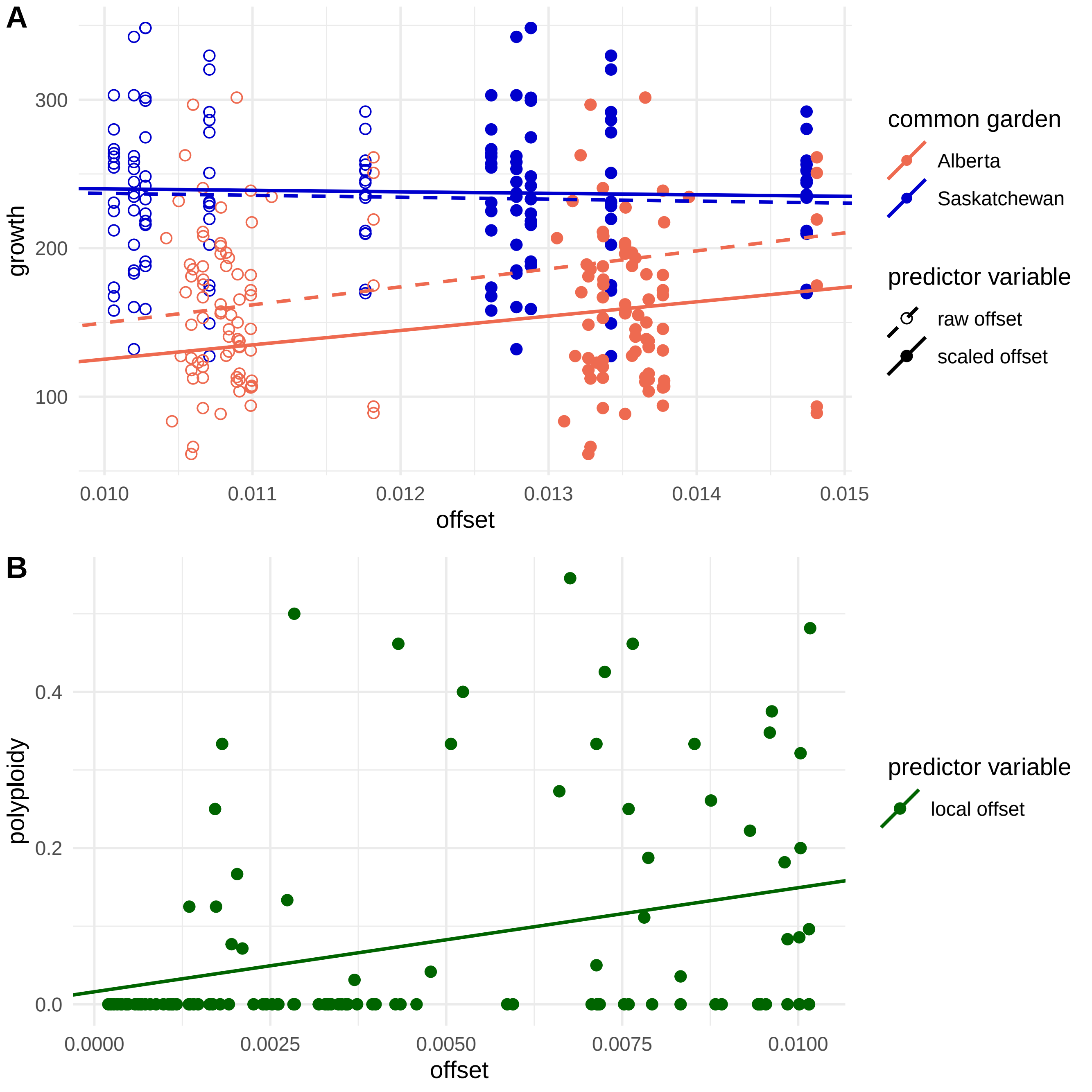
A
growth
offset
300
200
100
0.010
0.011
0.012
0.013
0.014
0.015
common garden
Alberta
Saskatchewan
predictor variable
raw offset
scaled offset
B
polyploidy
0.0
0.2
0.4
0.0000
0.0025
0.0050
0.0075
0.0100
offset
predictor variable
local offset
