# Supplementary material for: Assessing Future Maladaptation Risk in the Keystone Species Populus tremuloides
Source: Evol Appl. 2026 Oct 13;19(10):e70332. doi: 10.1111/eva.70332 (PMC13659280; doi:10.1111/eva.70332)
Supplement: Supplementary file 6 [file EVA-19-e70332-s008.pdf]

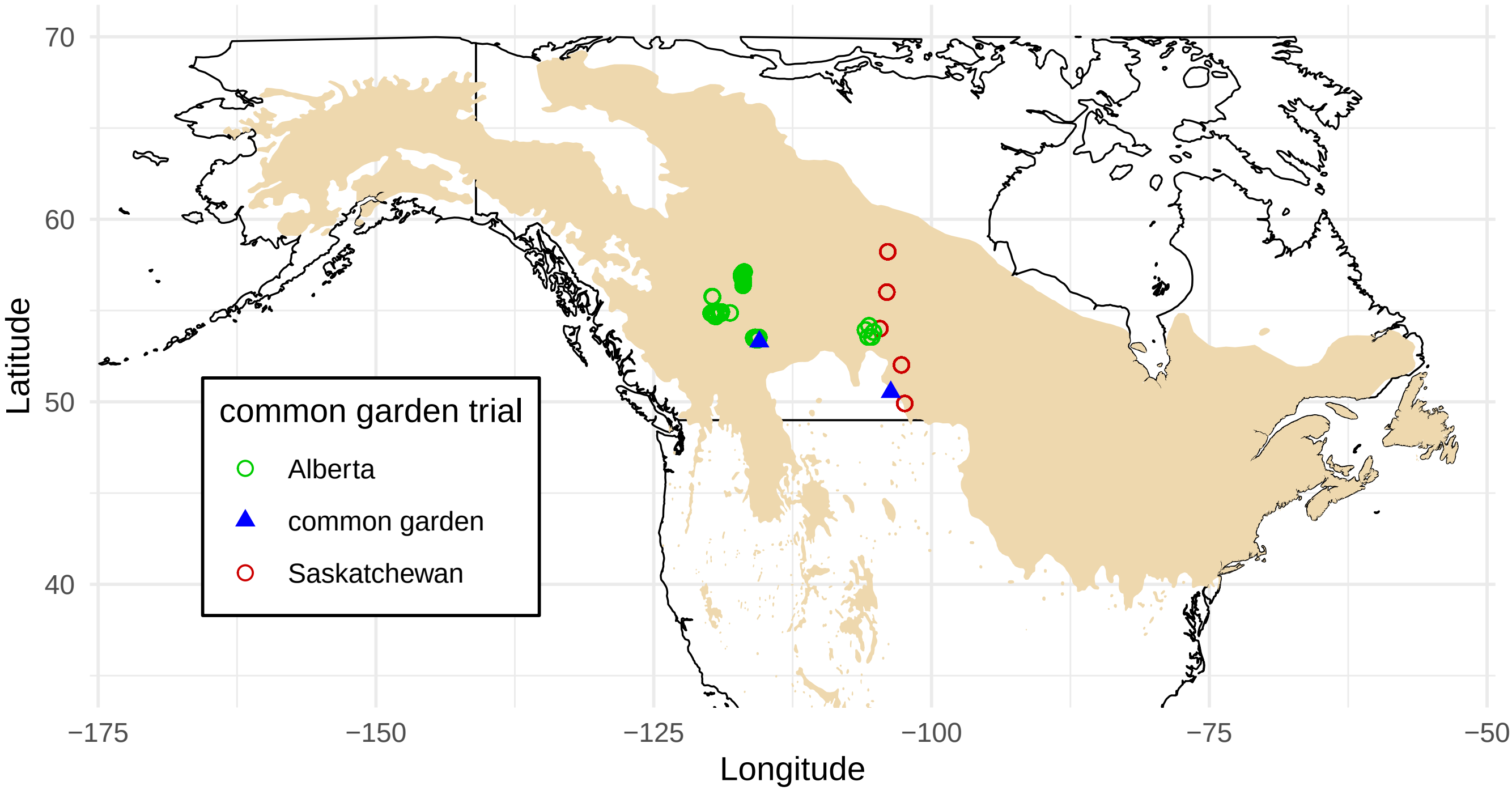

70
60
50
40
Latitude
−175
−150
−125
−100
−75
−50
Longitude
common garden trial
Alberta
common garden
Saskatchewan
