# Supplementary material for: Assessing Future Maladaptation Risk in the Keystone Species Populus tremuloides
Source: Evol Appl. 2026 Oct 13;19(10):e70332. doi: 10.1111/eva.70332 (PMC13659280; doi:10.1111/eva.70332)
Supplement: Supplementary file 7 [file EVA-19-e70332-s003.docx]

**Supplementary tables**

*Table S 1: Genetic cluster characteristics*

| **Genetic Cluster** | **Total Populations** | **Total Individuals** | **Avg. Distance Between Populations (km)** |
| --- | --- | --- | --- |
| NENA | 40 | 365 | 765.83 |
| NWNA | 24 | 199 | 709.80 |
| WUS | 22 | 120 | 722.09 |
| MX | 25 | 207 | 379.98 |

*Table S 2: Importance values for environmental variables for the different datasets*

| **Environmental Variable** | **Datasets** | | | |
| --- | --- | --- | --- | --- |
|  | Full | Neutral | Bayenv2 | LFMM |
| MEM1 | 0.004012 | 0.004339 | 0.012527 | 0.009539 |
| MEM2 | 0.003872 | 0.003995 | 0.004543 | 0.005961 |
| MEM3 | 0.007837 | 0.009835 | 0.006004 | 0.013028 |
| MEM4 | 0.004364 | 0.004879 | 0.009808 | 0.00952 |
| MEM5 | 0.004749 | 0.005161 | 0.004178 | 0.007625 |
| AHM | 0.004459 | 0.005121 | 0.004668 | 0.005209 |
| bFFP | 0.004475 | 0.005052 | 0.004211 | 0.006375 |
| CMD | 0.009645 | 0.011173 | 0.011477 | 0.012203 |
| CMI | 0.005844 | 0.006427 | 0.007574 | 0.007359 |
| DD_0 | 0.006897 | 0.007764 | 0.005892 | 0.008104 |
| DD_18 | 0.006136 | 0.0065 | 0.005802 | 0.00684 |
| DD1040 | 0.005217 | 0.005588 | 0.00527 | 0.005932 |
| DD18 | 0.003427 | 0.00361 | 0.003687 | 0.004094 |
| DD5 | 0.005612 | 0.00603 | 0.005689 | 0.006307 |
| eFFP | 0.003208 | 0.003484 | 0.003113 | 0.003861 |
| EMT | 0.004937 | 0.005163 | 0.004841 | 0.005796 |
| Eref | 0.010253 | 0.013983 | 0.007275 | 0.011812 |
| EXT | 0.00465 | 0.005627 | 0.004445 | 0.006831 |
| FFP | 0.004334 | 0.005068 | 0.004063 | 0.005541 |
| MAP | 0.004454 | 0.004799 | 0.005719 | 0.005617 |
| MAT | 0.003567 | 0.003724 | 0.003431 | 0.003999 |
| MCMT | 0.006913 | 0.007596 | 0.005971 | 0.008458 |
| MSP | 0.008153 | 0.009451 | 0.008545 | 0.012804 |
| MWMT | 0.002837 | 0.003197 | 0.003531 | 0.004022 |
| NFFD | 0.004366 | 0.004772 | 0.004014 | 0.004942 |
| PAS | 0.006004 | 0.007141 | 0.005508 | 0.006768 |
| RH | 0.003136 | 0.003711 | 0.002627 | 0.004092 |
| SHM | 0.007989 | 0.009113 | 0.007508 | 0.01411 |
| TD | 0.008537 | 0.00957 | 0.007588 | 0.011119 |

*Table S 3: SNPs selected to build the GF models for the different genetic cluster*

| **Genetic cluster** | **SNPs** | **Selected SNPs Model** | **Mean R^2^ (%)** | **Overlap with Dataset containing all individuals** |
| --- | --- | --- | --- | --- |
| All | 420 | 316 (75%) | 22 | - |
| NENA | 420 | 18 (4%) | 8 | 17 |
| NWNA | 420 | 12 (3%) | 12 | 10 |
| WUS | 420 | 137 (33%) | 16 | 119 |
| MX | 420 | 55 (13%) | 7 | 45 |

*Table S 4: Donor and recipient importance for LFMM dataset with climate scenario SSP3-7.0*

| **Region /**  **Genetic cluster** | **Donor Importance (%)** | | | **Recipient Importance (%)** | | |
| --- | --- | --- | --- | --- | --- | --- |
|  | median | min | max | median | min | max |
| Full Range | 76.13 | 0.01 | 80.35 | 74.36 | 0.00 | 86.47 |
| Boreal | 82.16 | 0.01 | 84.56 | 81.98 | 0.00 | 88.82 |
| Temperate | 68.77 | 0.02 | 78.21 | 66.63 | 0.00 | 85.23 |
| Subtropical | 37.54 | 0.46 | 49.85 | 38.08 | 0.00 | 58.15 |
| NENA | 70.88 | 4.09 | 80.7 | 67.64 | 0.00 | 89.87 |
| NWNA | 65.7 | 0.29 | 78.63 | 65.94 | 0.00 | 90.26 |
| WUS | 79.12 | 0.12 | 84.75 | 82.41 | 0.00 | 85.52 |
| MX | 48.13 | 1.07 | 86.63 | 48.66 | 0.53 | 88.24 |

*Table S 5: The liner model parameters for assessing the relationship between genomic offset and fitness (growth) or polyploidy.*

| **Response Variable** | **Predictor Variable^1^** | **Common Garden** | **Intercept** | **Slope** | **R^2^** | **p-Value** | **AIC** |
| --- | --- | --- | --- | --- | --- | --- | --- |
| Growth (volume) | raw offset | Alberta | 28.58 | 12115.53 | < 0.01 | > 0.05 | 834.21 |
|  | scaled offset |  | 28.59 | 9665.91 | < 0.01 | > 0.05 | 834.21 |
| Growth (height) | raw offset | Saskatchewan | 250.02 | -1309.13 | < 0.01 | > 0.05 | 774.29 |
|  | scaled offset |  | 250.02 | -1044.43 | < 0.01 | > 0.05 | 774.29 |
| Polyploidy | local offset | - | 0.01 | 13.32 | 0.1 | < 0.01 | -124.81 |

^1^ *Genomic offset parameter calculation described in methods section.*
